# Correlation of symptomatic enterovirus infection and later risk of allergic diseases via a population-based cohort study

**DOI:** 10.1097/MD.0000000000005827

**Published:** 2017-01-27

**Authors:** Zon-Min Lee, Ying-Hsien Huang, Shu-Chen Ho, Ho-Chang Kuo

**Affiliations:** a Department of Pharmacy, Kaohsiung Chang Gung Memorial Hospital, Taiwan; b Department of Pediatrics and Kawasaki Disease Center, Kaohsiung Chang Gung Memorial Hospital and Chang Gung University College of Medicine, Taiwan; c Department of Public Health, College of Health Sciences, Kaohsiung Medical University, Kaohsiung, Taiwan.

**Keywords:** allergic disease, cohort study, enterovirus

## Abstract

Infants who are exposed to the rhinovirus or respiratory syncytial virus are at a higher risk of subsequently developing wheezing or asthma. This study aims to determine whether preschoolers with a history of symptomatic enterovirus infection are at an increased risk of developing allergic diseases or not.

We used data from the Taiwan National Health Insurance Research Database from 1999 to 2006 for this nationwide population-based cohort study. The subsequent risks for allergic diseases, which included asthma (International Classification of Diseases [ICD]-9: 493.X), allergic rhinitis (AR; ICD-9 CM code 477.X), and atopic dermatitis (AD; ICD-9-CM code 691.X), were compared between herpangina (ICD-9: 074.0) and hand, foot, and mouth disease (HFMD; ICD-9: 074.3) throughout the follow-up period using the Cox proportional hazards model.

In this database, 12,016 neonates were born between January 1999 and December 1999. Among them, we further evaluated 8337 subjects; 3267 children infected with either herpangina or HFMD served as the study cohort, and the other 5070 children made up the comparison cohort. Children in the herpangina group had a higher risk of developing AR and AD, with adjusted hazard ratios of 1.15 (1.02–1.30, 95% CI) and 1.38 (1.17–1.63. 95% CI), respectively, while children suffered from HFMD had decreased risks of asthma, with an adjusted hazard ratio of 0.76 (0.63–0.93, 95% CI).

Children who previously suffered from herpangina experienced an increased risk of subsequently developing AD and AR. Meanwhile, children who had suffered from HFMD experienced a decrease in the subsequent occurrence of asthma compared to the general population.

## 1 Introduction

Previous studies have found that early rhinovirus-induced wheezing significantly influences childhood asthma.^[1]^ Asthma is often exacerbated by viral infections, which intensify allergic inflammation,^[2]^ a reaction caused mostly by the production and release of cytokines that subsequently leads to the recruitment and activation of type 2 innate lymphoid cells that secrete mediators.^[2]^ Furthermore, young children who have contracted severe bronchiolitis caused by the respiratory syncytial virus (RSV) are at a greater risk of subsequently developing asthma^[3]^; lower respiratory tract infections with the RSV and rhinovirus also significantly correlate with an increased occurrence of asthma.^[4]^ Enteroviruses, which are related to various human diseases, can often be found in young children, but the relationship between enteroviruses and the subsequent incidence of asthma or other allergic diseases is yet to be known.

Herpangina and hand, foot, and mouth disease (HFMD) are both infectious diseases caused by a variety of human enterovirus genotypes that are often present in preschool children.^[5]^ Herpangina is a febrile illness characterized by oral ulcerations and vesicular rashes that are predominantly located on the posterior oropharyngeal structures.^[6]^ Meanwhile, HFMD is a viral febrile illness that frequently presents as oral ulcerations on the anterior tonsillar pillars, soft palate, and buccal mucosa, as well as vesiculo-papular rashes on the feet, hands, elbows, knees, and buttocks.^[5]^ Various enterovirus species and genotypes, such as human enterovirus 71, Coxsackie virus A, Coxsackie virus B, etc., have been discovered to be etiological agents for HFMD and herpangina.^[5]^ In general, children who contract either one of these diseases often experience mild clinical symptoms that resolve several days after the infection.^[5]^ However, the positive identification of enterovirus 71 or myoclonic jerk and pleocytosis in the cerebrospinal fluid may be effective predictors of lesions on magnetic resonance imaging or unfavorable neurological sequelae, respectively.^[7]^

Allergic diseases are common and important health problems for children and have had an increasing burden on the general population in recent years.^[8,9]^ While an allergic disease rarely causes in death, its symptoms can persist for a long time and may negatively affect sufferers’ quality of life. As a result, understanding how allergic diseases develop and resolve throughout infancy and childhood is vital to clarifying their pathophysiology.^[8]^ Tang et al^[10]^ found that the incidence of allergic diseases, like asthma, atopic dermatitis (AD), and allergic rhinitis (AR), has radically increased in various countries during the past 20 to 30 years. This escalation has been prompted by numerous factors, including the increased use of antibiotics, the consumption of sterilized foods, improved hygiene, and smaller family sizes, all of which have contributed to less contact with germs and decreased childhood infection rates.^[11]^ This trend has also been caused by the increased use of materials or products that emit volatile organic compounds.^[12]^

Besides the aforementioned environmental factors that have spurred the unprecedented growth of allergic disorders, previous research has found that a host's immune response and invading pathogens also play a vital role.^[13]^ A virus-specific antibody-secreting B cells response has been identified in the first week of illness in children infected with genotype B5 enterovirus 71.^[14]^ Furthermore, a lower antiechovirus antibody response was found in children admitted to hospitals with asthma exacerbation.^[15]^ Other research observed the increased frequency of circulating follicular helper T cell observed in children who have HFMD caused by an enterovirus 71 infection.^[16]^ However, regarding whether the enterovirus-affected immune system affects the subsequent occurrence of asthma, AD, or AR has yet to be determined.

## 2 Methods

As population-based studies related to enteroviruses and the subsequent risks of developing asthma, AR, and AD have not previously been reported for PubMed review, we performed the following procedures to establish the relationship between enteroviruses in young children and the incidence of later development of an allergic disease.

### 2.1 Data source

The Taiwan's National Health Insurance (NHI) program, which provides comprehensive health insurance, was first implemented on March 1, 1995. More than 98% of Taiwan's 23 million residents have received various types of healthcare services under the NHI, including Chinese medicine, inpatient and outpatient care, dental care, physical therapy, childbirth, etc.^[17]^ The Bureau of NHI randomly sampled a substitute database of 1,000,000 subjects from all of its registrants and released an insurance data set to the public for research purposes which the present study used. Furthermore, the National Health Research Institute (NHRI) has stated that no statistically significant differences have been found with regard to healthcare costs, age, or gender between the selected subjects and all enrollees in the NHI program.^[17]^

These NHRI databases have been used for various purposes, such as diagnostic information, prescription use, hospitalization, and epidemiological research, all of which are of high quality. Adopting one of the aforementioned databases, this study was exempt from full review by Chang Gung Memorial Hospital's the Institution Review Board (No. 102-0364B) since the identification numbers of the patients in the database had previously been encrypted to protect their privacy.^[11]^

### 2.2 Study cohort

This study comprises an independent birth cohort. Since HFMD and herpangina cases surged due to the outbreak of enterovirus infections in 1998 in Taiwan,^[18]^ we chose to study 1999, during which 12,016 infants were born according to the birth dates listed in the enrollment data files from the Longitudinal Health Insurance Database 2000. Any children with a history of asthma (International Classification of Diseases [ICD]-9 CM code 493.X), AR (ICD-9 CM code 477.X), or AD (ICD-9-CM code 691.X) before a diagnosis of herpangina (ICD-9 CM code 074.0) or HFMD (ICD-9 CM code 074.3) were excluded. Furthermore, children with a history of asthma, AR, or AD within the first 2 years of life, that is, 1 to 2 years of age, or children who had experienced any 1 of these 3 diseases within 1 year after a herpangina or HFMD infection were also excluded. Ultimately, we included a total of 8337 children in the cohort study group.

In this study, children with a symptomatic enterovirus infection (herpangina or HFMD) were defined as those who had been sick with such infection within the first 5 years of life. The subsequent occurrence of asthma, AR, and AD was followed from the moment that the herpangina or HFMD was diagnosed until 2005, which is the end of this study's follow-up period. To explore the relationship between these allergic diseases and certain comorbidities, we also examined the effects of prematurity and low birth weight, that is, a gestational age of <37 weeks and a birth weight of <2500 g, respectively.

### 2.3 Statistical analysis

We used SAS version 9.3 (SAS Institute, Cary, NC) for data analysis. Children affected in the first 2 years of life were excluded, and then the incidence rates of AD, asthma, and AR among the remaining children were calculated separately within the 1999 birth cohort. The prevalence rates of AD, asthma, and AR were distinctly calculated for children ages 2 to 6 years. Cox proportional hazards regression analysis, stratified by gender and medical diseases, was carried out. The development of outcomes observed among the study's participants was followed until they were 6 years old. Hazard ratios (HRs) and the corresponding 95% confidence intervals (CIs) were determined using the group that had neither herpangina nor HFMD to serve as the reference group.

## 3 Results

According to Taiwan's nationwide database, a total of 12,016 infants were born between January 1999 and December 1999, 1281 of them suffered from asthma, 1981 from AD, and 417 from AR, without ever having been diagnosed with herpangina or HFMD. The aforementioned children were excluded, so this study consists only of the 8337 children who matched the inclusion criteria. Of those, the 3267 children who suffered from either herpangina or HFMD which we made the study cohort, and the other 5070 children made up the comparison cohort. Table 1 shows the demographic data of both the study and control subjects, divided by the presence or absence of herpangina or HFMD. Regarding confounding factors, prematurity and low birth weight, that is, a gestational age of <37 weeks and a birth weight of <2500 g were found to have a significant influence (*P* = 0.0005). We found that diseases like asthma, AD, and AR are correlated with herpangina or HFMD.

**Table 1:**
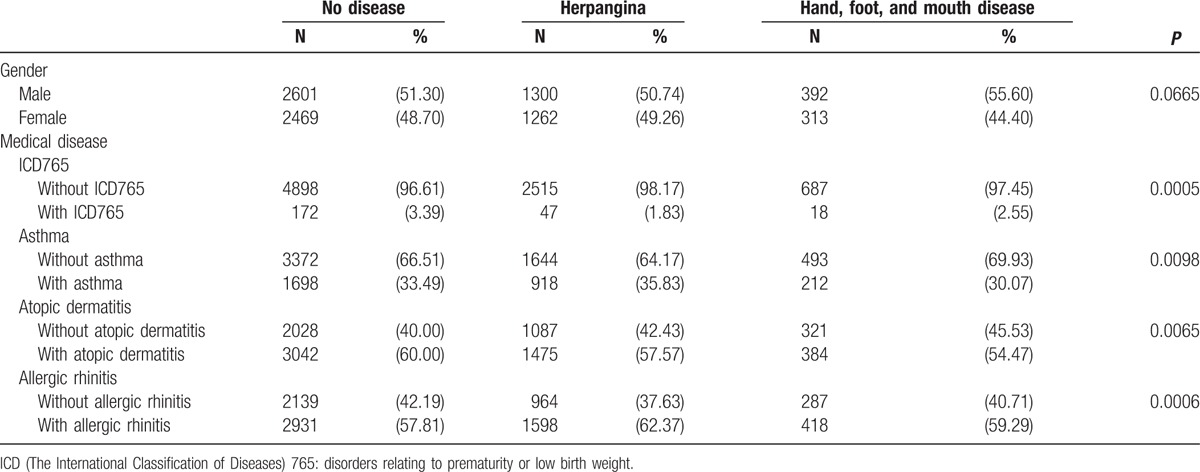
Demographic characteristics of the study and comparison groups, stratified by the presence or absence of herpangina or hand, foot, and mouth disease between 1999 and 2003.

**Figure 1:**
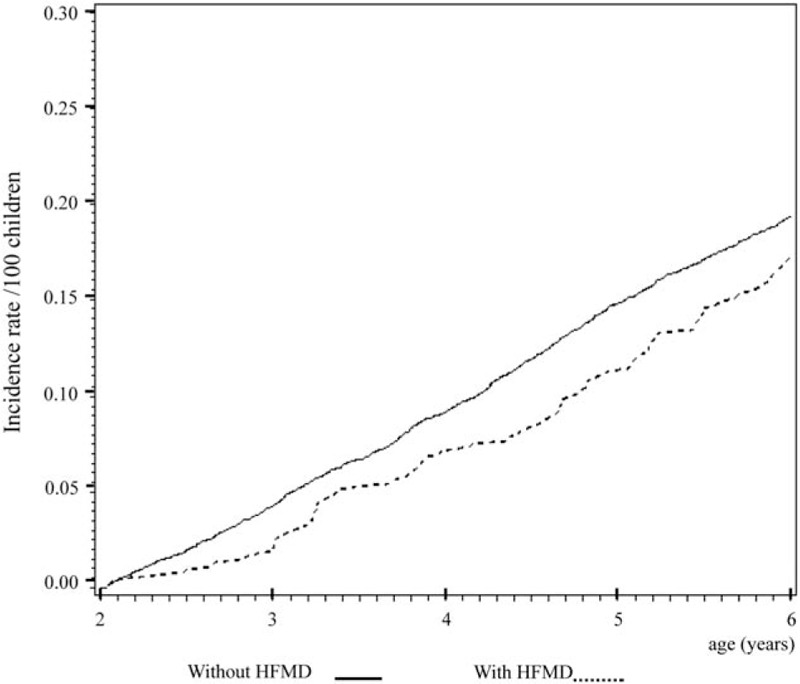
Incidence rate of allergic rhinitis lower in hand, foot, and mouth disease group than controls.

**Figure 2:**
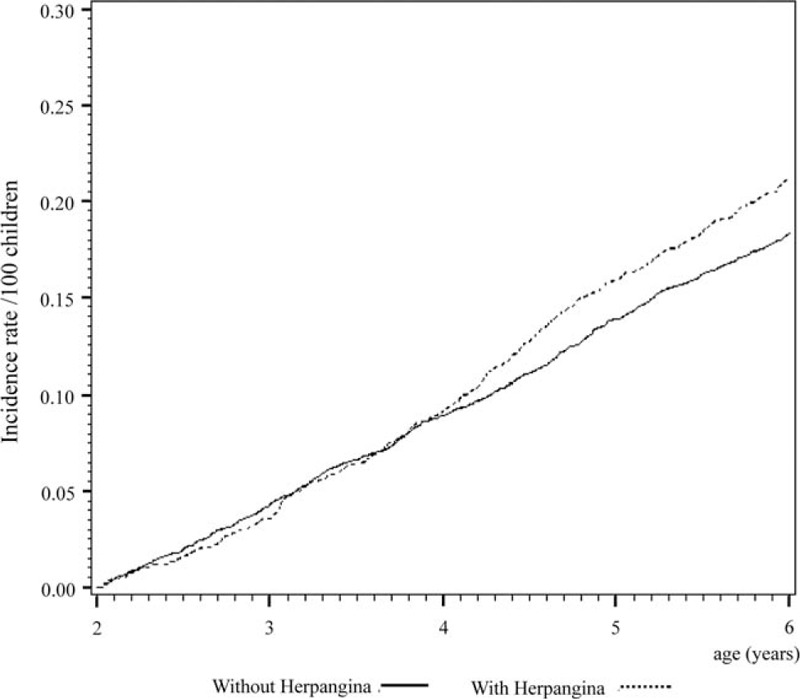
Incident rate of allergic rhinitis increased around 4 years old in the herpangina group.

**Figure 3:**
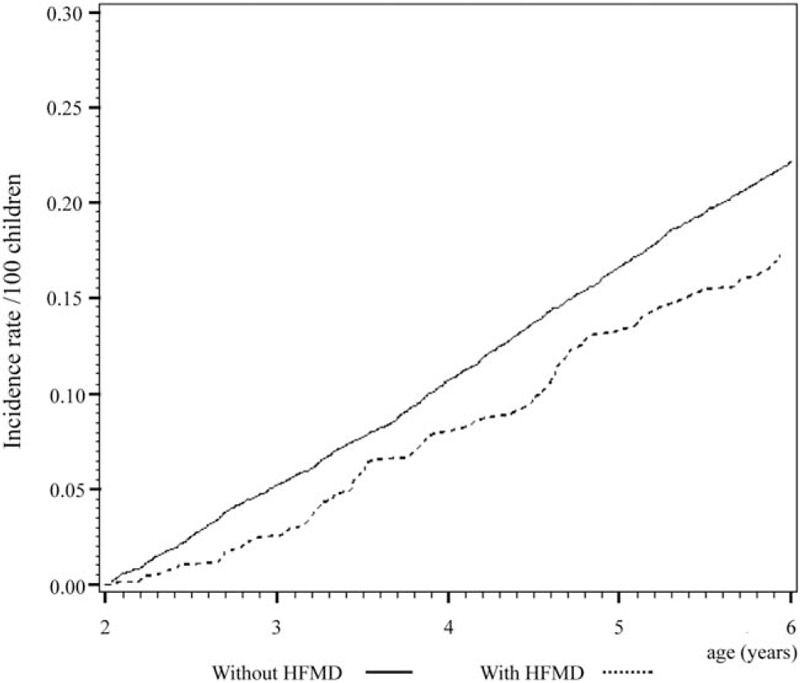
Incidence rate of asthma was found to be lower in hand, foot, and mouth disease group throughout the whole follow-up period.

**Figure 4:**
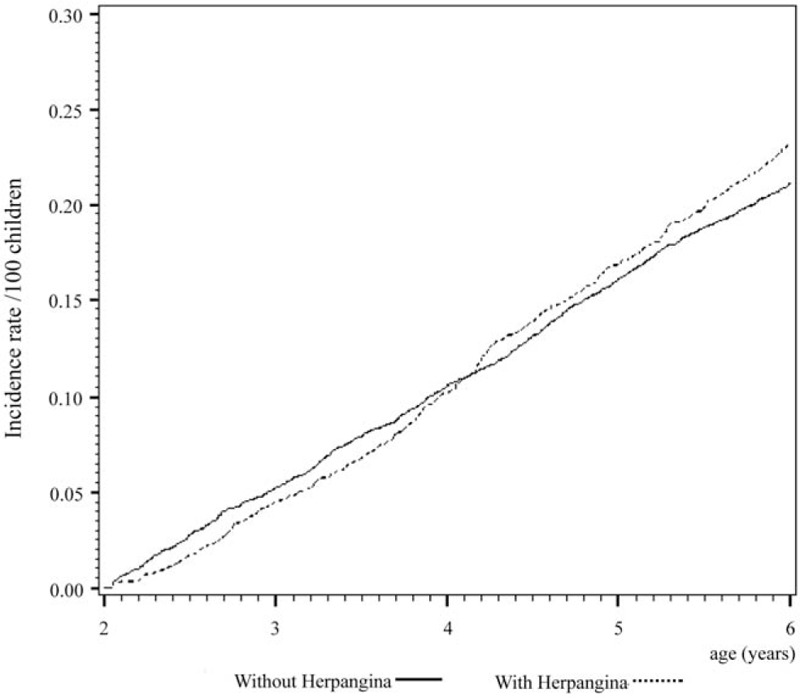
Incident rate of asthma increased around 4 years old in the herpangina group.

**Figure 5:**
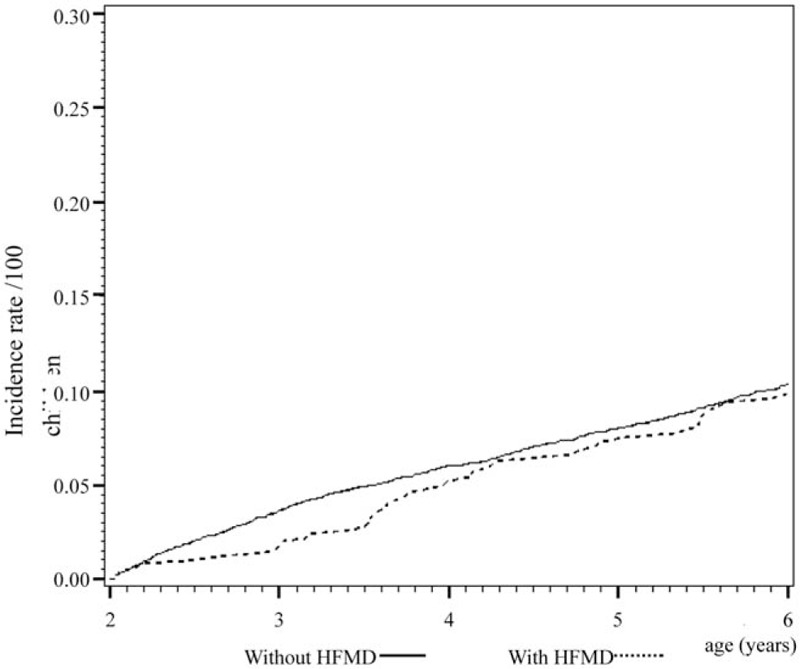
Incidence rate of atopic dermatitis lower in hand, foot, and mouth disease group throughout the whole follow-up period.

**Figure 6:**
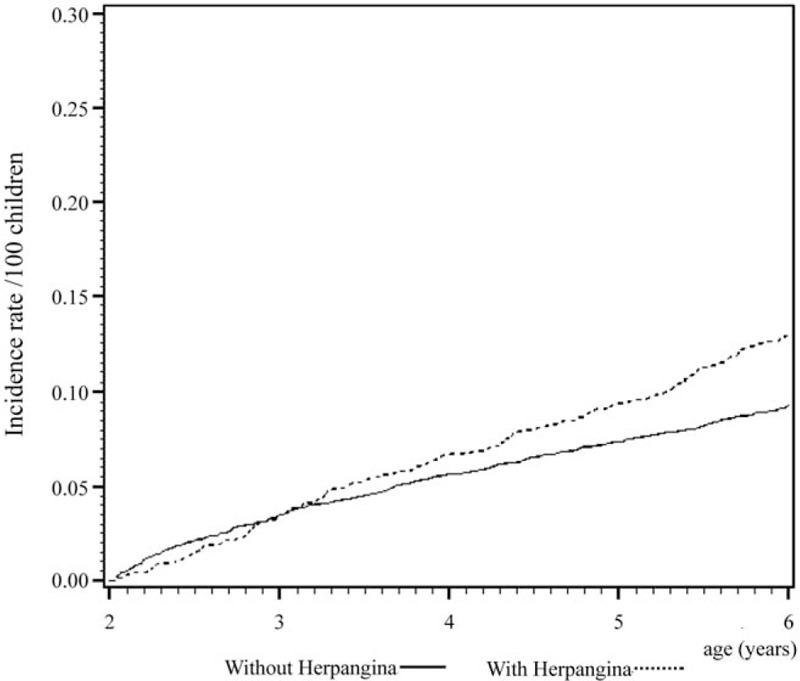
Incidence rate of atopic dermatitis increased around 3 years old in the herpangina group.

Cox regression analysis of the temporal relationship between the moment infected with herpangina or HFMD and the subsequent development of the 3 examined allergic diseases in the 1999 birth cohorts is shown in Figs. 1 to 6. The incidence rates of the 3 examined atopic diseases (AR, Fig. 1; asthma, Fig. 3; AD, Fig. 5) were found to be lower in the HFMD group throughout the whole follow-up period while the incident rates of AR (Fig. 2) and asthma (Fig. 4) increased around 4 years old and AD (Fig. 6) increased around 3 years old in the herpangina group.

### 3.1 Children with a previous diagnosis of HFMD were at a lower subsequent risk of developing asthma

As shown in Table 2, asthma was found in 105 out of 664 (15.8%) children with HFMD and 1342 out of 6751 (19.9%) children who had not been infected with this disease. Cox regression analysis showed that the HR for those children with HFMD remained significant even after making adjustments for potential confounders (adjusted HR: 0.76; 95% CI 0.63–0.93, *P* < 0.05) throughout the 5-year follow-up period; that is, children with HFMD were at a lower risk for the later development of asthma. Children who had suffered from HFMD showed no significant difference with those who had not been ill with said disease with regard to the risk of developing subsequent AR and AD, *P* > 0.05.

**Table 2:**
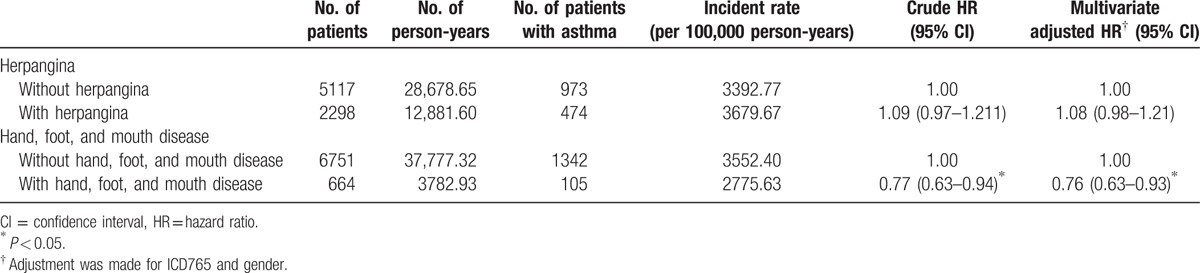
Rates and hazard ratios of asthma of preschool children infected with herpangina or hand, foot, and mouth disease in comparison with those in the control cohort during the 5-year follow-up period.

### 3.2 Children with a previous diagnosis of herpangina were at a higher subsequent risk of AR

AR was found in 395 out of 2074 (19.0%) children with herpangina and 826 out of 4934 (16.7%) children who had not been infected with this disease. Cox regression analysis showed that the HR for children with herpangina remained significant even after making adjustments for potential confounders (adjusted HR: 1.15; 95% CI 1.02–1.30, *P* < 0.05); that is, children with herpangina were at a higher risk of developing AR (Table 3).

**Table 3:**
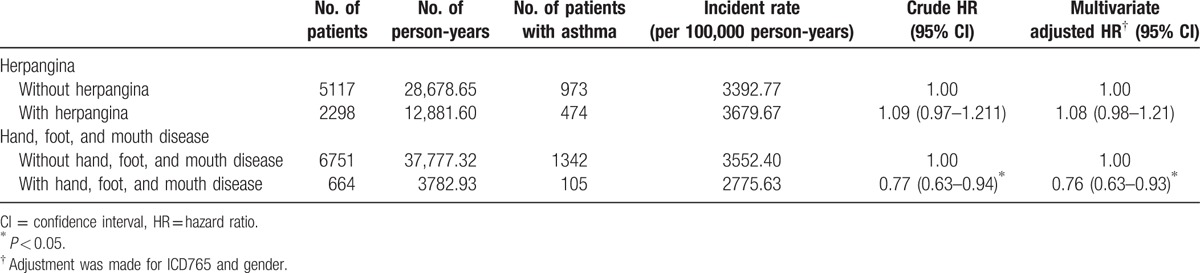
Rates and hazard ratios of allergic rhinitis of preschool children infected with herpangina or hand, foot, and mouth disease in comparison with those in the control cohort during the 5-year follow-up period.

### 3.3 Children with a previous diagnosis of herpangina were at a higher subsequent risk of AD

AD was found in 213 out of 1762 (12.1%) children with herpangina and 386 out of 4356 (8.9%) children who had not been infected with this disease. Cox regression analysis showed that the HR for children with herpangina remained significant even after making adjustments for potential confounders (adjusted HR: 1.38; 95% CI 1.17–1.63, *P* < 0.05); that is, children with herpangina were at a higher risk of developing AD (Table 4). Regarding asthma, no significant difference was found after children suffered from herpangina, *P* > 0.05.

**Table 4:**
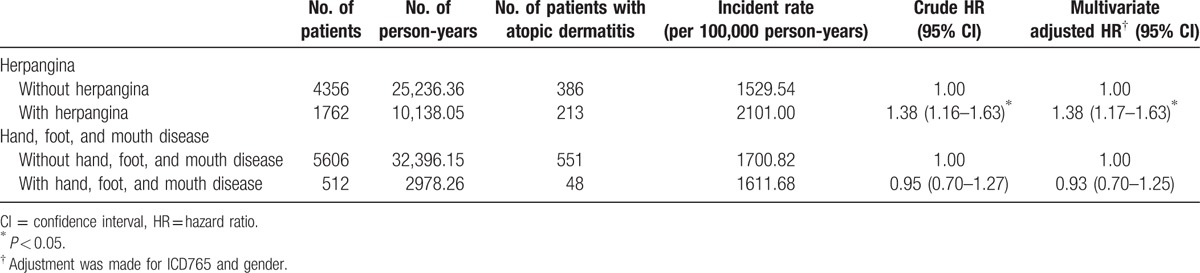
Rates and hazard ratios of atopic dermatitis of preschool children infected with herpangina or hand, foot, and mouth disease in comparison with those in the control cohort during the 5-year follow-up period.

## 4 Discussion

Infants who have previously suffered from a rhinovirus, rhinovirus-induced lower respiratory tract infection, or RSV are at an increased risk of developing asthma later in life.^[1,3,4]^ Furthermore, asthma is often exacerbated by viral infections that increase allergic inflammation.^[2]^ Immunity is also important with regard to the development or increased risk of certain allergic diseases. According to Tsukagoshi, human enterovirus infections may be correlated with virus-induced asthma^[19]^ but no population-based research has yet proven this hypothesis. The present study is the first population-based cohort study with a nationwide dataset and 5-year follow-up period to explore the relationship between enteroviruses and the subsequent development of allergic diseases in children. This study's results show that young children who have suffered from an enterovirus infection, depending on the disease, may either have an increased or decreased risk of subsequently developing certain allergic disease in comparison to the control subjects.

We recruited children infected with symptomatic herpangina or HFMD within the first 5 years of life to explore the subsequent risks of allergic diseases because these 2 infectious diseases are commonly caused by an enterovirus in young children^[5]^ particularly in children under the age of 5.^[20]^ Furthermore, previous studies have found lower antiechovirus antibody responses in children with exacerbated asthma^[15]^ and an increased frequency of circulating follicular helper T cells in children with HFMD.^[16]^ This finding suggests that enteroviruses may affect the immune system in such a way that influences the subsequent risks of allergic diseases. Moreover, this study only included children infected with symptomatic herpangina or HFMD since asymptomatic sufferers most likely did not seek medical treatment.

In part due to neonatal respiratory morbidity, premature births have been associated with an increased risk of asthma-like symptoms,^[21]^ while both low birth weight and prematurity are significantly correlated with the decreased occurrence of AR in male conscripts.^[22]^ Furthermore, a low birth weight indicates a protective factor for the risk of AD.^[23]^ Ullemar et al^[24]^ reported that children born with a low gestational age or low birth weight have an increased risk of developing asthma. Therefore, prematurity or low birth weight may significantly influence the risk of the subsequent development of allergic diseases; these 2 factors are presented as ICD 765 and have been adjusted in this study.

An immune response of changing EV71 seropositive patterns was observed in children infected with herpangina or HFMD^[25]^; immune responses have also been identified in animal models to be vital with regard to protection from enterovirus 71 infection.^[16]^ Adaptive immune reaction-pathogen and antigen-specific responses can occur after either a herpangina or HFMD infection, but the timing for immunity development has not yet been clearly defined and varies among young children. Therefore, children diagnosed with asthma, AR, or AD within 1 year after a herpangina or HFMD infection were excluded from this study to reduce the number of cases not influenced by an enterovirus infection.

Enteroviruses are related to chronic inflammatory and autoimmune diseases.^[26]^ Cytokines, which are small proteins fundamental in cell signaling, manage the balance between immune responses and promote the Th1 immune response and antiviral process.^[27]^ Humoral mediators like cytokines are a crucial part of the pathophysiology of viral infections,^[27]^ and some research has suggested that cytokines in particular contribute to EV71 pathogenesis. Furthermore, the interaction between EV71 and receptors may be correlated with the cytokines immunopathogenesis.^[28]^ Cytokines, including IFN-γ, TNF-α, IL-4, IL-6, and IL-10 levels, have been correlated with HFMD severity^[29]^; thus, the production of cytokines corresponds with the severity and stage of clinical symptoms and signs, in addition to the infection's source.^[30]^

This study's results revealed that herpangina and HFMD have differing influences on the subsequent risk of developing an allergic disease. Therefore, these enterovirus-induced diseases, the former characterized by fever and oral ulcers, and the latter by fever with oral ulcerations and vesicular eruptions on the skin,^[5]^ apparently vary in immunomodulation and are distinct in their cytokine production.

The results found herein are quite unique; children with herpangina had a higher subsequent risk of developing AR and AD, while those with HFMD were at a lower risk of subsequent asthma development when compared to the general population (see Section 3). According to a previous study presented by Ye et al,^[31]^ plasma levels of the pro-inflammatory cytokines IL-1β and IL-6 were considerably higher in children who were severely ill with enterovirus 71 than both mildly ill children and noninfected controls; this finding indicates that IL-1β and IL-6 production correlates with the enterovirus-induced disease's severity. These 2 interleukins are pro-inflammatory cytokines, so a more severe disease produces more pro-inflammatory cytokines. Compared to children in the herpangina group, those infected with HFMD have more serious symptoms and supposedly have more pro-inflammatory cytokines. This may lead to comparably lower anti-inflammatory cytokines and thus a decreased risk of allergic diseases. As predicted, children infected with HFMD have a relatively lower risk of developing asthma, AR, and AD in this study, with an adjusted HR of 0.76 (95% CI 0.63–0.93), 0.88 (95% CI 0.71–1.08), and 0.93 (95% CI 0.70–1.25), respectively.

In patients with AD, lymphocytes that infiltrate early lesioned skin reveal a Th2 pattern of cytokine secretion, mainly IL-4 and/or IL-13, as well as the typical Th2-type chemokine receptor CCR4,^[32]^ which indicates that AD is a Th2 skewing disease. This finding may explain in part why the risk of AD was highest among the 3 investigated allergic diseases in both cohorts. With regard to asthma, Th2 cells are mostly predominant in mild to moderate asthma and thus do not solely cause the development of severe asthma,^[33]^ which implies that factors besides Th2 cells play a more important role in the development of this disease. These findings may partly explain the lower risk of asthma in both cohorts (see Section 3).

This study had several limitations. First, analysis of urbanization and geographic regions was not performed, but we are certain that such data may have some biases; therefore, the results in this study do not fully reflect the real world. Second, we explored the subsequent risks of asthma, AR, and AD with and without the presence of herpangina or HFMD only during the 5-year follow-up period in young children, instead of the entire childhood period. Third, the detailed mechanism for immunity change or how an immune system modified by an enterovirus may influence the subsequent risk of developing an allergic disease is still unclear. Fourth, the effects of diseases other than herpangina and HFMD that are caused by enterovirus infections have yet to be determined. Fifth, neither young children infected with rhinovirus or RSV, nor who had a family history of allergic disease was adjusted for in this study. Despite these limitations, the nationwide database provided information on a large sample size, and adjusting for certain covariates related to these allergic diseases was relatively objective with regard to determining the relationship between enteroviruses and the subsequent incidence of allergic diseases in children during the 5-year follow-up period in this study.

## 5 Conclusion

This study is the first to investigate the risk of developing allergic disease in children after having herpangina and HFMD by using a population-based study, as well as the first to find that herpangina and HFMD differ with regard to their effect on subsequent allergic disease development. While herpangina and HFMD are both caused by enterovirus infections, but each 1 influences the subsequent risks of developing an allergic disease differently. These 2 diseases apparently vary in immunomodulation, with herpangina, which is characterized by fever with oral ulcers, increasing the subsequent risk of AD and AR, and HFMD, which is more severe and generates more pro-inflammatory cytokines, decreasing the prevalence of asthma later in life when compared to the general population. Additional studies are warranted to explore the mechanism of changes in the immune system caused by an enterovirus influencing the subsequent development of allergic diseases in humans.
